# Manganese-enhanced MRI (MEMRI) in breast and prostate cancers: Preliminary results exploring the potential role of calcium receptors

**DOI:** 10.1371/journal.pone.0224414

**Published:** 2020-09-15

**Authors:** Gabriella Baio, Marina Fabbi, Michele Cilli, Francesca Rosa, Simona Boccardo, Francesca Valdora, Sandra Salvi, Luca Basso, Laura Emionite, Eliana Gianolio, Silvio Aime, Carlo Emanuele Neumaier

**Affiliations:** 1 NNUH-Norfolk and Norwich University Hospital and Norwich Medical School, University of East Anglia, Norwich, United Kingdom; 2 Diagnostic Imaging and Senology Unit, IRCCS Ospedale Policlinico San Martino, Genoa, Italy; 3 Biotherapies Unit, IRCCS Ospedale Policlinico San Martino, Genoa, Italy; 4 Animal Facility, IRCCS Ospedale Policlinico San Martino, Genoa, Italy; 5 Lung Cancer Unit, IRCCS Ospedale Policlinico San Martino, Genoa, Italy; 6 Department of Health Sciences, University of Genoa, Genoa, Italy; 7 Department of Pathology, IRCCS Ospedale Policlinico San Martino, Genoa, Italy; 8 Department of Molecular Biotechnologies and Health Sciences & Molecular Imaging Center, University of Torino, Torino, Italy; National Institute of Radiological Sciences, QST, JAPAN

## Abstract

**Procedures:**

To preliminary assess the relationship between Manganese Enhanced Magnetic Resonance Imaging (MEMRI) and the expression of calcium receptors in human prostate and breast cancer animal models.

**Methods:**

NOD/SCID mice were inoculated with MDA-MB-231 breast cancer cells and prostate PC3 cancer cells to develop orthotopic or pseudometastatic cancer animal models. Mice were studied on a clinical 3T scanner by using a prototype birdcage coil before and after intravenous injection of MnCl_2_. Assessment of receptor’s status was carried out after the MR images acquisition by immunohistochemistry on excised tumours.

**Results:**

Manganese contrast enhancement in breast or prostate cancer animal models well correlated with CaSR expression (p<0.01), whereas TRPV6 expression levels appeared not relevant to the Mn uptake.

**Conclusion:**

Our preliminary results suggest that MEMRI appears an efficient tool to characterize human breast and prostate cancer animal models in the presence of different expression level of calcium receptors.

## Introduction

Regulation of calcium metabolism is crucial for cellular activities such as proliferation, gene transcription and cell death [1, 2]. To maintain a balanced condition between the intra- and extra-cellular compartments, cells employ specialized calcium pumps, channels and calcium binding proteins (the so called “molecular toolkit”) [3, 4]. Changes in calcium metabolism are reported on the upsurge of certain pathological states, including cancer [5–7]. To better detect the occurrence of an altered calcium metabolism *in vivo*, a non-invasive method such as imaging could be extremely valuable. On the basis of the close analogy between calcium and manganese, an imaging technique such as Manganese Enhanced Magnetic Resonance Imaging (MEMRI) has received increased attention in preclinical cancer imaging studies [8–10].

MEMRI is a well-established method that, by combining the peculiar physical and biological properties of Mn^2+^, allows to extract relevant anatomical and functional imaging information in a variety of systems [11–14].

Previously we tested MEMRI as imaging tool to report on the high expression levels of Calcium Sensing Receptor (CaSR) in a human breast cancer animal model [15]. CaSR is a G protein coupled receptor, which plays an important role in tumour development [16–19]. Breast tumours displaying high CaSR expression, as assessed by immunohistochemistry, correlated well with manganese uptake assessed by Magnetic Resonance Imaging (MRI). However, CaSR is not the only receptor involved in the presence of the altered calcium metabolism in cancer cells, as other calcium receptors or channels can interfere with Ca^2+^ homeostasis. Calcium-permeable ion channels, such as Transient Receptor Potential (TRP) have been recently associated with tumour progression [20]. An altered expression level of TRP channels is associated with changes in intracellular Ca^2+^ and in the proliferative pathways, promoting or inhibiting apoptosis of the tumour cells [21, 22]. Specifically, TRPV6 is a highly Ca^2+^-selective channel [23–26] and its activity is also known to be modulated by oestrogen, progesterone, tamoxifen and vitamin D, affecting proliferation and survival of cancer cells [27]. In prostate and breast cancer, TRPV6 is substantially associated with highly proliferative and metastatic tumours [27, 28]. A high level of expression of TRPV6 is associated with high Gleason score tumours and high metastatic risk [29, 30], supporting its potential use to predict the clinical outcome of human prostate cancer [31]. Similarly, in oestrogen receptor-negative breast cancer, high TRPV6 levels were associated to decreased survival rates when compared to patients with low or intermediate TRPV6 expression [32].

Considering the important role that TRPV6 and CaSR have in cancer, we undertook these pilot experiments to investigate the MEMRI response in different types of breast and prostate cancer animal models, characterized by different level of expression of these molecules.

## Results

To investigate whether the uptake of Mn^2+^ was correlating with different CaSR and TRPV6 expression levels, several murine models have been prepared, namely human breast orthotopic model, human prostate orthotopic model, intraosseous human breast model and human pseudometastatic prostate model. For each model, after the acquisition of the MEMRI experiment, the animals were sacrificed and the tumour cells analyzed for assessing the level of expression of CaSR and TRPV6, respectively. In all the experiments performed, shortly after the MRI session, the mice were euthanised using the carbon dioxide method, and especially before the onset of signs of distress and when the tumour reached the maximum diameter of 1 cm.

### Human breast orthotopic cancer animal model

**Fig 1:**
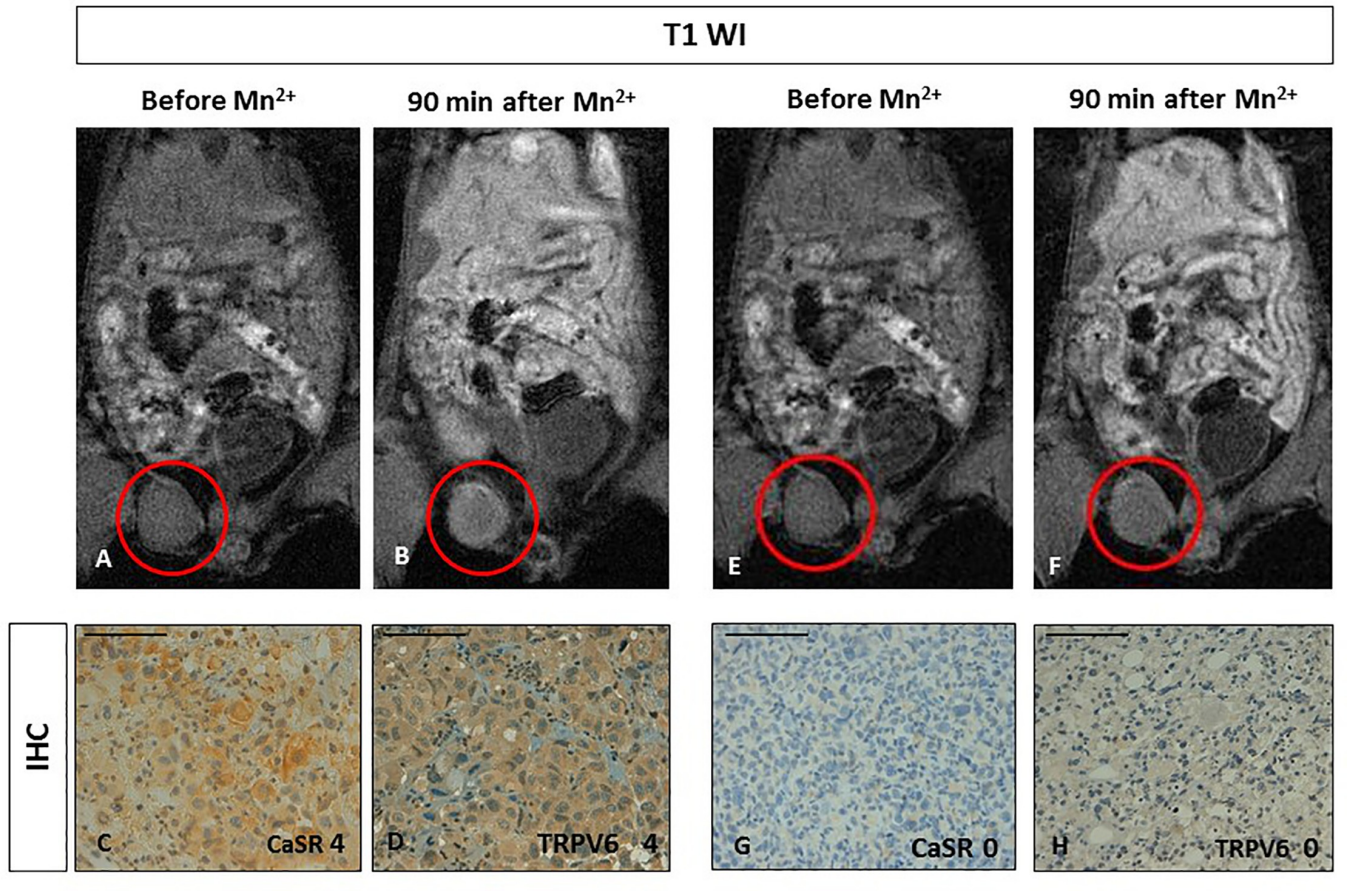
Manganese Enhanced MR Imaging (MEMRI) of orthotopic human breast cancer xenografts and CaSR/TRPV6 receptors levels. **AB.** Tumour diameter 5 mm. Upon comparing T1-weighted gradient echo images (T1 WI) recorded before and 90 minutes after Mn^2+^ administration, the tumour showed manganese uptake (red circles). **CD.** Immunohistochemistry (IHC) of CaSR and TRPV6: both receptors are detected (score 4) with a homogenous intense staining. Scale bar = 100μ. **EF.** Tumour diameter 5 mm. Upon comparing T1-weighted gradient echo images (T1 WI) recorded before and 90 minutes after Mn2+ administration, no changes in tumour signal enhancement was appreciated (red circles). **GH.** Immunohistochemistry (IHC) showed no evidence for CaSR and TRPV6 expression (score 0). Scale bar = 100μ.

MDA-MB-231 breast cancer cells were inoculated into NOD-SCID mice (n = 4) and MR images were acquired starting from week 6 after the cells transplant. T_2W_-MR imaging showed a rounded, well defined, lesion within the mammary fat-pad without any sign of infiltration of the surrounding tissue. The dynamics of manganese uptake resulted quite different among the considered mice. In two mice, 10 minutes after manganese administration, a slight peripheral contrast enhancement was observed in the tumour region, while a homogeneous contrast enhancement was gradually appreciated at later time points (30, 60 and 90 minutes) (Fig 1A and 1B). Quantitative analysis demonstrated an increased tumour signal enhancement (SE) of 91% at 90 min from the Mn^2+^ administration (see Table 1). In the other two mice, no manganese uptake was observed at all-time points (Fig 1E and 1F) as witnessed by the quantitative analysis that demonstrated no significant changes in SE (see Table 1).

**Table 1:** SNR and SE% (calculated as [(SNR-SNR^0^)/ SNR^0^]×100) for all tumour lesions respectively, of xenotransplant and pseudometastatic tumour animal model, before and 90 minutes after manganese administration and CaSR or TRPV6 expression levels evaluated by immunohistochemistry.

**Xenotransplant lesions**	**Before SNR**	**After Mn**^**2+**^ **SNR**	**SE %**	**CaSR score**	**TRPV6 Score**
Orthotopic PC3	7,61	7,64	+0.4	0	0
Orthotopic PC3	4,90	5,27	+7.5	1	1
Orthotopic MDA-MB-231	5,99	5,87	-2.0	0	0
Orthotopic MDA-MB-231	6,15	11,75	+91	4	4
Osseous MDA-MB-231	10,11	9,35	-7.5	0	0
**Pseudo-metastatic lesions (PC3)**	**Before SNR**^**0**^	**After Mn**^**2+**^ **SNR**	**SE %**	**CaSR score**	**TRPV6 score**
Liver nodule	11,28	10,98	-2.6	1	4
Liver nodule	14,52	13,70	-5.6	1	4
Mediastinal nodule	11,13	14,85	+33	3	2
Mediastinal nodule	11,48	13,41	+17	2	2
Diaphragmatic nodule	8,97	13,15	+47	3	4
Peritoneal nodule	11,82	11,77	-0.4	1	1
Peritoneal nodule	13,76	13,64	-0.9	1	1

To correlate manganese-enhancement with CaSR or TRPV6 expression, immunohistochemistry of the excised lesions was performed. As shown in Fig 1C, 1D, 1G and 1H, in these tumours the expression levels of CaSR and TRPV6 ranged between score 0 and 4 and both calcium receptors appear expressed at the same level in the same tumour. Interestingly, the CaSR or TRPV6 score levels correlated well with MEMRI SE observations.

### Human prostate orthotopic cancer animal model

**Fig 2:**
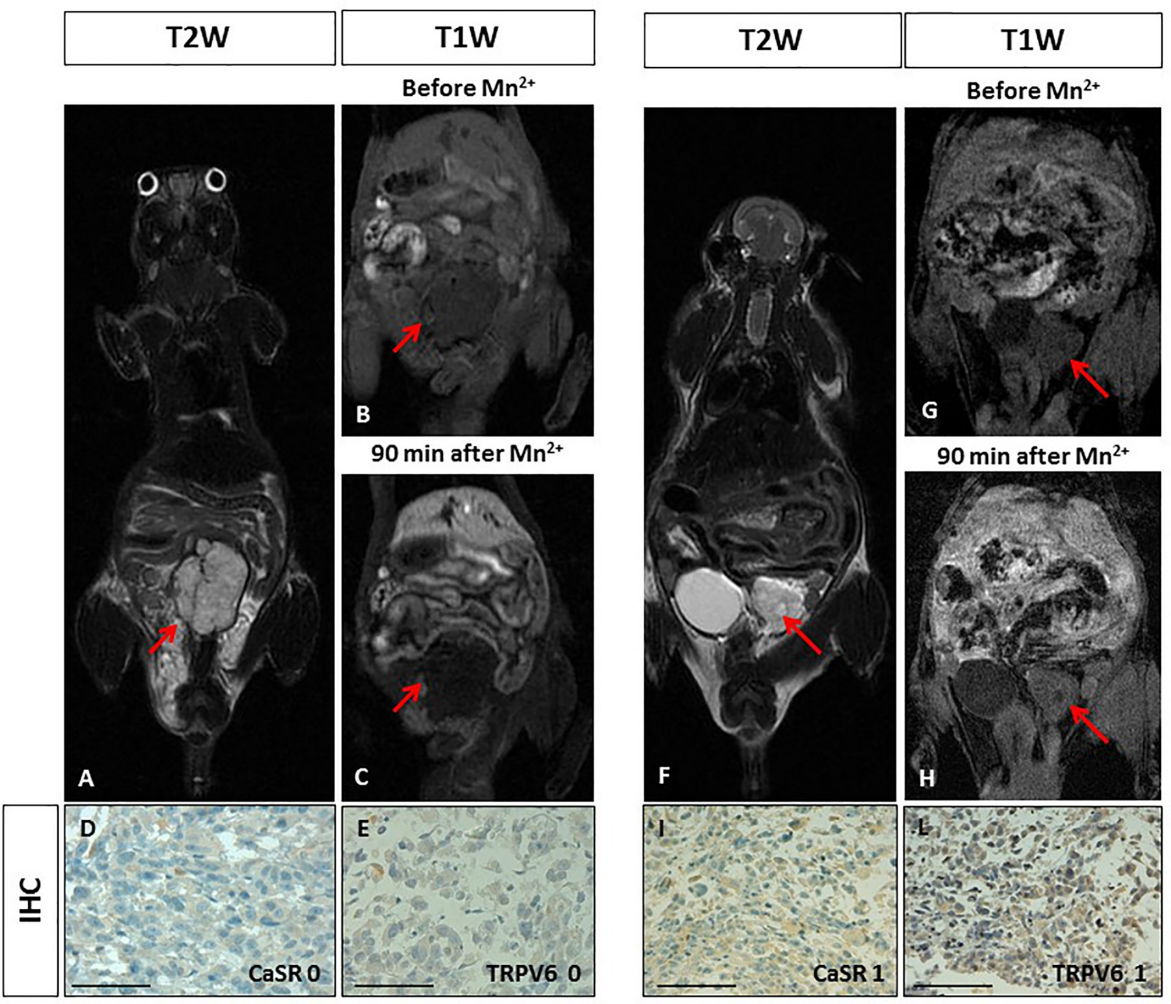
Manganese Enhanced MR Imaging (MEMRI) of orthotopic human prostate cancer xenografts and CaSR/TRPV6 receptors levels. Tumour diameter 10 mm. On T2-weighted MR image a high signal intensity multilobulated prostatic tumour is detected (red arrow). **BC.** On T1-weighted gradient echo MR images (T1 WI) recorded before and 90 minutes after Mn^2+^ administration, no changes in tumour signal enhancement was appreciated (red arrows). **DE.** Immunohistochemistry (IHC) of CaSR and TRPV6: both receptors were not detected in tumour cells (score 0) showing a negative staining. Scale bar = 100μ. **F.** Tumour diameter 6 mm. On T2-weighted image a high signal intensity extra-capsular prostatic tumour is detected (red arrow). **GH.** On T1-weighted gradient echo images (T1 WI) recorded before and 90 minutes after Mn2+ administration, no changes in tumour signal enhancement was appreciated (red arrows). **IL.** Immunohistochemistry (IHC) of CaSR and TRPV6: both receptors displayed a very low positive staining in tumour cells (score 1). Scale bar = 100μ.

The orthotopic prostate xenotransplant model (n = 2) was prepared by implantation of PC3 cells in the prostate gland tissue under sterile surgical conditions. T_2W_-MR images showed well-defined lobulated lesions in both animals after 6 weeks (Fig 2A and 2F). No manganese enhancement was detected within both tumours at MR visual analysis at all acquisition time points (10, 30 and 90 minutes) (Fig 2B, 2C, 2G and 2H). The quantitative analysis showed no significant difference in SE before and after manganese administration in both animals (SNR_beforeMn+_ 7.61 vs SNR_afterMn+_ 7.64; SNR_beforeMn+_ 4.90 vs SNR_afterMn+_ 5.27; see Table 1). The immunohistochemistry analysis of prostate tumours demonstrated a very low expression level of CaSR and TRPV6 with a score between 0 and 1 (Fig 2D, 2E 2I and 2L). The level of expression of both receptors was consistent in the same tumour.

### Intraosseous human breast cancer xenotransplant model

**Fig 3:**
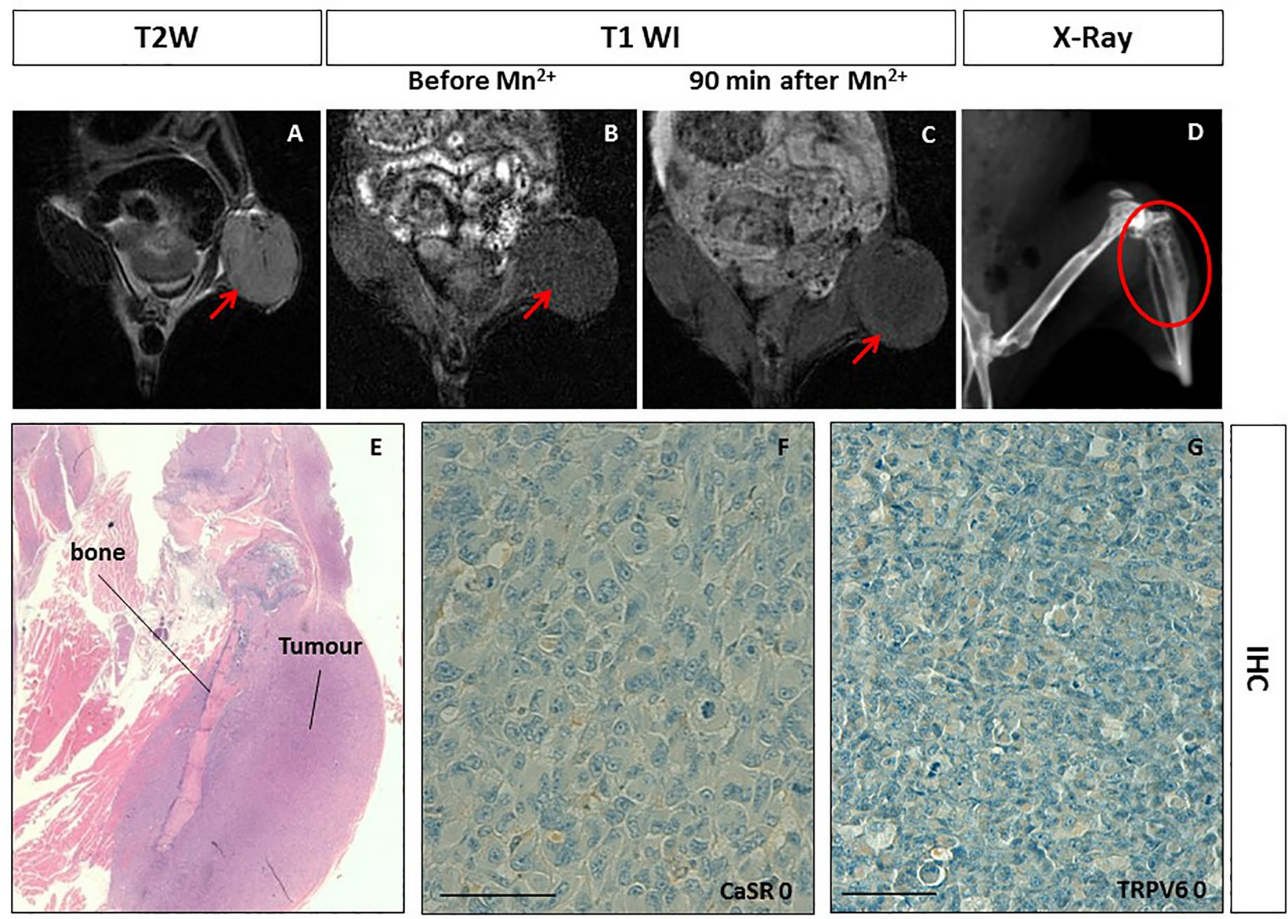
Manganese Enhanced MR Imaging (MEMRI) of the intraosseous human breast cancer xenotransplant model and CaSR/TRPV6 receptors levels. **A.** Tumor diameter 10 mm. On T2-weighted MR image a low signal intensity solid tumor is detected at the left leg of the mouse (red arrow). **BC.** On T1-weighted gradient echo MR images (T1 WI) recorded before and 90 minutes after Mn^2+^ administration, no changes in tumor enhancement was appreciated (red arrows). **D.** X-Ray imaging of the left leg of the mouse showed multiple lytic areas within the tibia in keeping with the tumor transplantation (red circle). **E.** Hematoxylin and eosin staining displaying the intraosseous tumor. **FG.** Immunohistochemistry (IHC) of CaSR and TRPV6: both receptors were not detected in tumor cells (score 0) showing a negative staining. Scale bar = 100μ.

To investigate the manganese uptake in pseudo-metastatic bone breast cancer lesions, the intraosseous human breast cancer xenotransplant model (n=1) was used. The osseous cancer model was set up according to Corey et al. [33]. As shown in Fig 3, T2 weighted MR images of the bone xenotransplant appeared as a well-defined soft rounded lesion in the left-leg (Fig 3A and 3E). Radiographic examination showed a cortical swelling of the tibia and tiny lytic lesions (Fig 3D). Upon manganese administration, on T1-weighted images, no contrast enhancement was observed within the tumour mass at all acquisition time points (Fig 3B and 3C) as confirmed by the quantitative analysis (see Table 1). The immunohistochemistry analysis showed no CaSR and TRPV6 (Fig 3F and 3G) expression.

### Human pseudometastatic prostate cancer animal model

**Fig 4:**
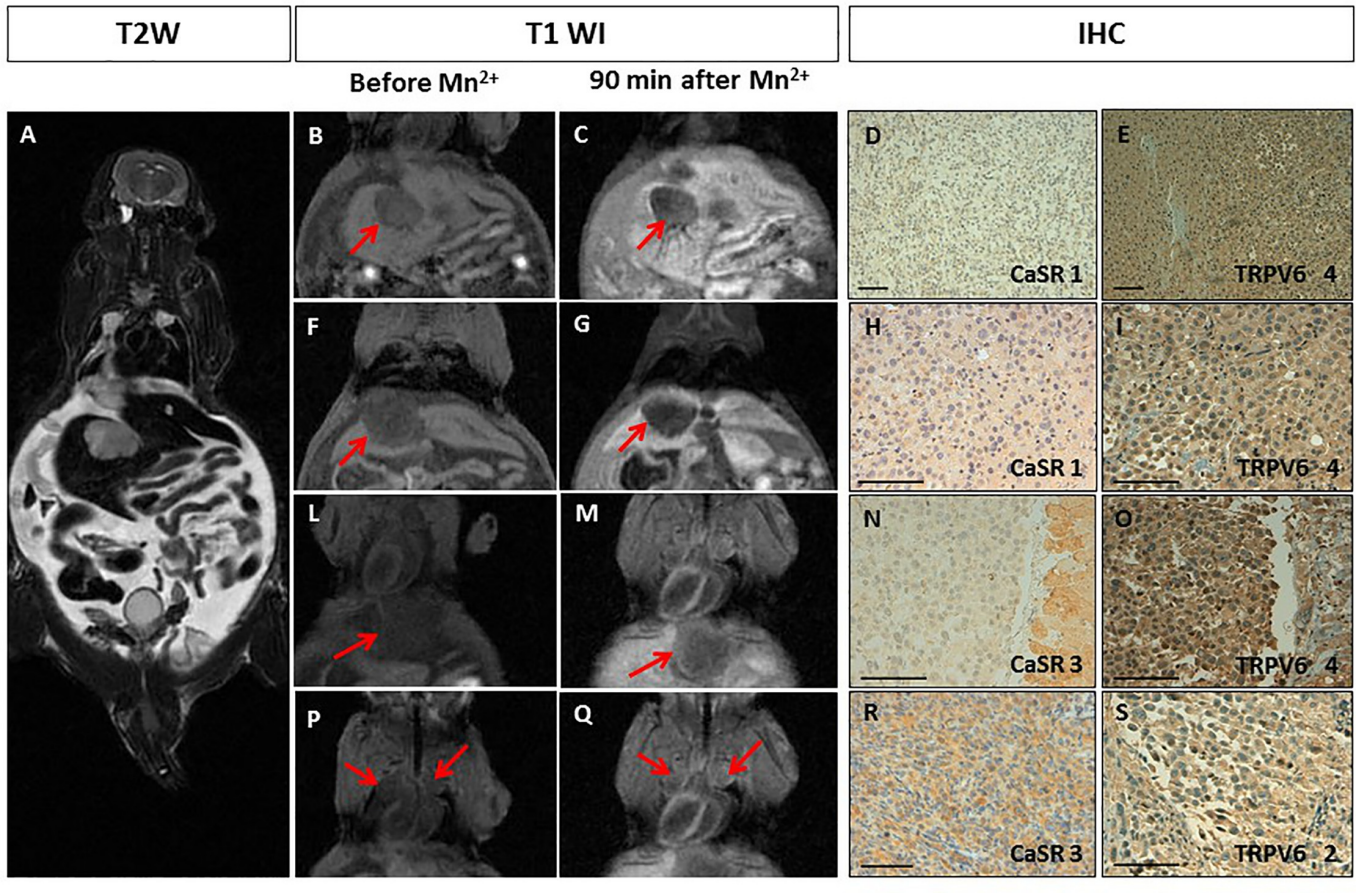
Manganese Enhanced MR Imaging (MEMRI) of the pseudometastatic prostate cancer animal model and CaSR/TRPV6 receptors levels. **A.** Multiple tumours with diameter from 3 to 10 mm. T2-weighted MR images showed multiple high intensity nodules with intra-abdominal ascites. **B-Q.** T1-weighted gradient echo MR images (T1 WI) recorded before and 90 minutes after Mn^2+^ administration: **B-G.** No manganese uptake by the liver metastases was detected (red arrows); **L-Q.** An increase in signal enhancement after manganese administration, respectively, within a diaphragmatic (LM) and mediastinal nodules (PQ) is appreciated (red arrows). **D-S.** Immunohistochemistry (IHC) of CaSR and TRPV6: **D-I.** A rare positive staining of CaSR was detected in tumour cells (score 1), while TRPV6 receptors displayed intense staining (score 4); **N-S.** Non-uniform weak/intense expression of CaSR was detected in tumour cells (score 3), while TRPV6 receptors ranged from strong uniform (score 4) to rare positive (score 2). Scale bar = 100μ.

**Fig 5:**
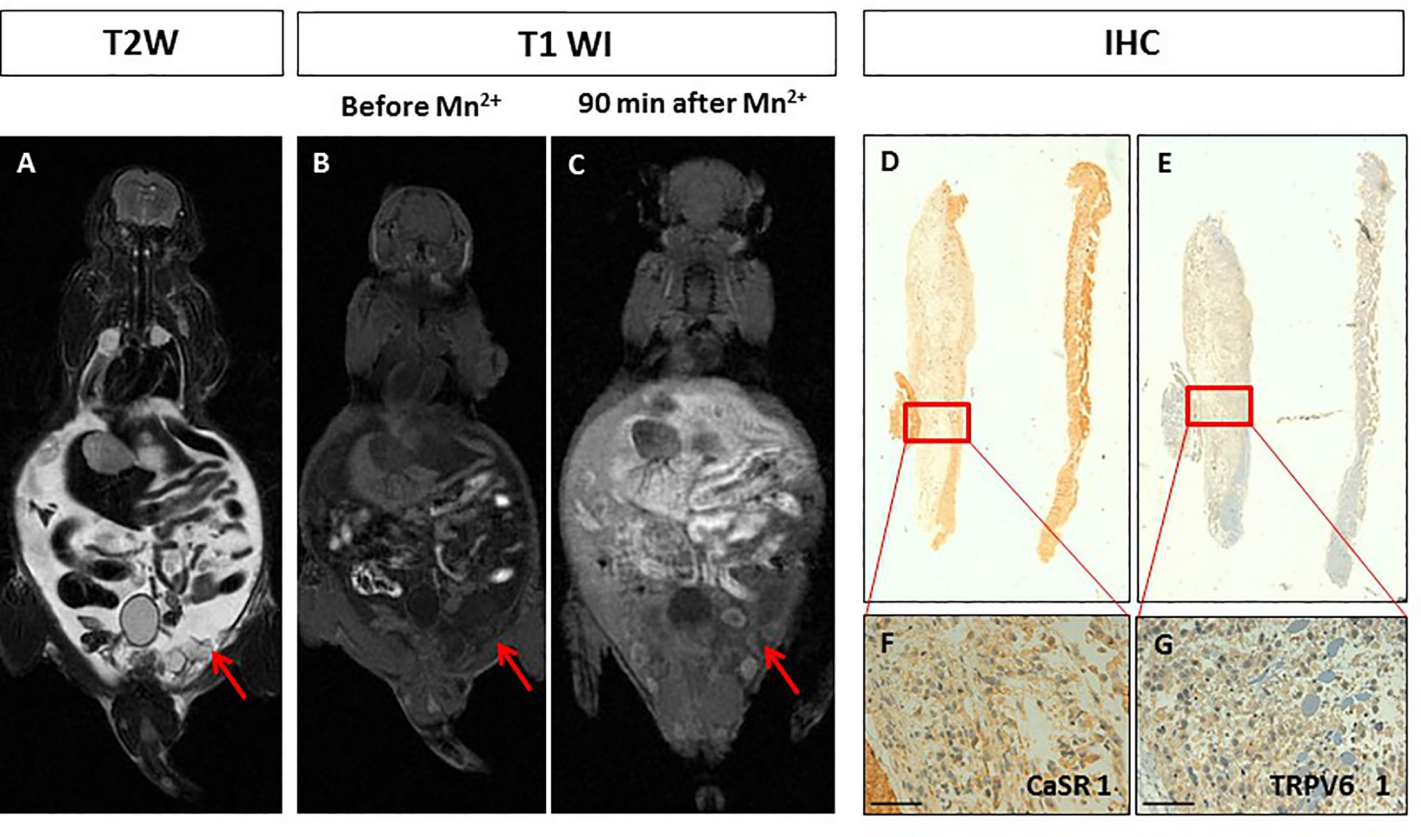
Manganese Enhanced MR Imaging (MEMRI) of pseudo-metastatic prostate cancer animal model and CaSR/TRPV6 receptors levels. **A.** Multiple peritoneal nodules with diameter of 3 mm (the same mouse of Fig 4). In T2-weighted MR image multiple high intensity peritoneal nodules with ascites are detected. **BC.** This slice shows the peritoneal nodules in T1-weighted gradient echo images (T1 WI) recorded before and 90 minutes after Mn^2+^ administration. No manganese uptake is detected (red arrows); **D-G.** Immunohistochemistry (IHC) of CaSR and TRPV6: A rare positive staining of both receptors was detected in tumour cells (score 1). Scale bar = 100μ.

The pseudometastatic prostate cancer animal model (n = 2), obtained by intra-cardiac injection of PC3 cells, developed multiple lesions in the mediastinum, liver and peritoneal deposits with ascites, respectively (Figs 4A and 5A). One of the pseudometastatic cancer animal model progressed quickly and MEMRI was not carried out (results are showed in S1 Fig in S1 File). After 90 min, the prostatic lesions showed different manganese uptake (from peripheral to full contrast enhancement) which remained stable for 3 hours (Figs 4B–4Q, 5B and 5C). Quantitative analysis demonstrated a large heterogeneity in the SE of all the metastatic deposits (SE range from -0.9 to 47% (see Table 1). Different expression levels of both calcium receptors (ranging from 0 to 3 for CaSR and from 0 to 4 for TRPV6) was observed, in particular, in some of the prostatic lesions the expression levels of the two calcium receptors were different (Figs 4D–4S and 5D–5G).

## Discussion

Several studies demonstrated the important role of calcium channels in carcinogenesis and in the modulation of cancer progression by inducing high metastatic risk [34–37]. Thus, it was deemed important to explore the use of non-invasive imaging methods, to better investigate these receptors and their relationship with cancer development. In preclinical cancer research, additional noninvasive evaluation methods that are reliable and easily performed are needed. The development of high-resolution in vivo imaging technologies provides a unique opportunity for studying disease in real time, in a quantitative manner, at the molecular level, along with the ability to repeatedly and non-invasively monitor disease progression or response to treatment. The greatest advantages of preclinical imaging techniques include the reduction of biological variability and the opportunity to acquire unique information along with the substantial reduction in the number of animals required for a particular study.

In our previous study, we applied MEMRI to image breast cancer tumours with a high-level expression of CaSR [15]. Calcium sensing receptor is associated with cancer cell proliferation and bone metastatic risk in breast cancer patients [16, 17]. In the present work, we expanded the study to Transient Receptor Potential channels (TRP) that are widely expressed both in normal tissue and cancer [20]. Among all TRP-channels, TRPV6 is a highly Ca^2+^-selective channel and therefore quite distinguishable especially in Ca^2+^-related intracellular pathways.

We used MDA-MB-231 breast and PC3 prostate cancer cell lines normally expressing both CaSR and TRPV6 at high levels. However, in *in vivo* cancer animal models, these tumour cells displayed different expression levels (score 0-4) for both calcium receptors. Different manganese uptake was observed in the corresponding MR images. Orthotopic human breast tumours showed either absence (score from 0) or high-level expression (score 4) of both receptors at the immunohistochemistry analysis, while in the orthotopic human prostate tumours expression of both receptors was low or absent (score 0-1). Despite these data should be considered preliminary, they indicate that manganese uptake observed at MR imaging was in accordance with the level of expression of both receptors (Figs 1 and 2). Certainly, more experiments are needed to confirm our results.

The different expression level (score 0-4) of calcium receptors observed in *in vivo* was not expected, since these cancer cell lines are expressing high level of both receptors. The mechanism leading to the “presence or absence” of these receptors in tumour is unclear. In our previous clinical studies, we demonstrated a different CaSR level expression (score 0-4) in human primary breast cancers [38], in keeping with other clinical studies that further correlated the different expression level of CaSRs to the tumour proliferation and, in turn, to high risk of bone metastases [37].

The orthotopic breast or prostate cancer animal model results are suggesting, at a first glance, a potential combined role of both calcium receptors in tumour manganese uptake. Previous in vitro studies demonstrated the link between CaSR and TRPV6, where TRPV6 action induces calcium ions entry in CaSR [39, 40]. The potential leading role of calcium receptors in manganese uptake has also been recently demonstrated by Castets CR et al, in a study where breast or glioma cancer cells lines pre-treated with Mn^2+^ and then incubated with calcium blockers enabled to retain Mn^2+^ ions into the cells [41].

Metastatic cancer animal models are very challenging due to the reproducibility of invasiveness of the models; moreover, they showed to recapitulate the intra and inter-tumour heterogeneity usually observed in human metastatic tumours (bone and visceral metastases). We developed intraosseous human breast cancer animal models (for bone metastasis) and metastatic prostate cancer animal models (for visceral metastases) and investigated the role of MEMRI in these types of cancer animal models. The intraosseous human breast cancer animal models did not show manganese uptake in keeping with the quantitative analysis of SE. SE changes at MEMRI well correlated with both calcium receptors status detected by immunohistochemistry (CaSR and TRPV6 score 0). In these models, MEMRI and receptors expression levels behaved analogously to what observed in the orthotopic breast or prostate cancer animal models.

It is worth of notice that the two mice with metastatic prostate cancer developed visceral metastases at very different rates. On the mouse that progressed quickly only immunohistochemistry was performed (S1 and S2 Figs in S1 File). MEMRI was performed on the second mouse and heterogeneous manganese uptake in all the visceral metastatic deposits was observed with a SE% that ranged from -0.9 to 47%. In this case the expression level of CaSR and TRPV6 receptors was markedly different, instead of what observed for the other cancer animal models. TRPV6 was highly expressed in most of the metastatic deposits with a score 4 (Fig 4) while very low-level expression of CaSR (score 0-1) was detected, except for diaphragmatic and mediastinal nodules with a CaSR score 3. All the metastatic nodules with low level of CaSR did not show any manganese uptake (Table 1) even when TRPV6 was highly expressed, while the diaphragmatic/mediastinal nodules with the higher expression level of CaSR (score 3) showed good manganese uptake with a SE% of 33-47%.

**Fig 6:**
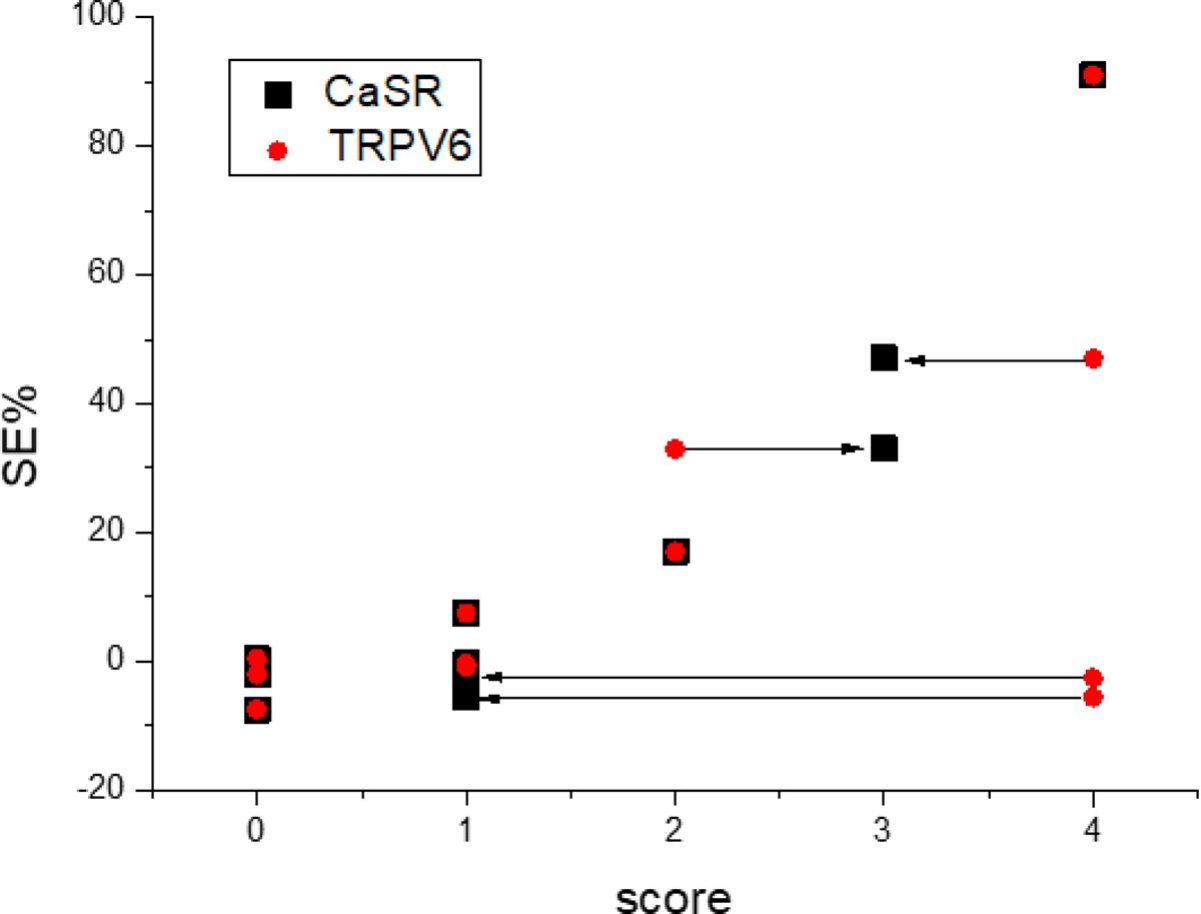
Dependence of MR signal enhancement (SE%) from the score of CaSR (black square) and TRPV6 (red circles) expression. The Mn-induced enhancement increases with CaSR expression; the expression level of TRPV6 appears not relevant for the increase of the MR signal.

Overall, as shown in Fig 6, the obtained results suggest a potential link between CaSR and manganese uptake. Manganese ions are analogues of calcium ions and cellular uptake of the two ions is expected to occur through the same mechanism(s) [42]. However, we observed (Fig 6) that only CaSR correlates with the cellular uptake of Mn^2+^ ions. The SE observed at MRI (i.e. 90 min after i.v. administration) could be the result of the transfer of manganese ions from the extracellular to the intracellular compartment, through the action of CaSR receptors. The absence of CaSR or its low-level expression resulted in undetectable MEMRI effects. In the liver, TRPV6 was highly expressed in both the healthy tissue and the metastatic deposits, even though normal liver tissue displayed manganese uptake while liver metastatic deposits did not.

One cannot exclude that other mechanisms rather than calcium receptors, are leading or affecting tumour manganese uptake. For example, use of ketamine as anaesthetic in our experiments might have affected TRPV6 calcium entry and thus, manganese uptake. However, we did not observe any abnormalities in manganese uptake by liver or kidneys, which express high levels of this type of calcium receptor.

The data available from the literature, are all different and sometimes with contradictory results. Other groups explored routes to target tumour with manganese; however, the applied methods differ drastically in term of the timing between Mn^2+^ administration and MRI detection. In some studies, it was 24 hours [43], while in our experiments we observed manganese uptake at shorter times (e.g. 30, 60, 90 min until 3 hours). Moreover, the contribution of perfusion to Mn^2+^ accumulation cannot be completely ruled out. Indeed, although Mn^2+^ is a free ion, its blood half-life is nearly 2 hours long [44], therefore within the 90 minutes time-frame, tumour regions with high vascularity, permeability and perfusion could be reached by a continuous flux of Mn^2+^ and retain it with the contribution of CaSR-mediated uptake. The overall accumulation of Mn^2+^ could thus be due to the combined effect of perfusion due to the level of tumour vascularity and CaSR activity.

Another important technical aspect affecting not only tumour uptake, but also increasing the toxicity of the contrast agent, is the concentration of Mn^2+^ administered. In all experiments, we used the same concentration of manganese below the toxicity threshold and, still allowed to achieve good signal enhancement by MR [45]. Furthermore, we cannot exclude the possibility that different tumour vascularization between prostate and breast cancers, might affect manganese uptake and thus, its distribution during the first minutes after injection. Further studies are needed to demonstrate the relationship between tumour vessel density-calcium receptor expression level and manganese uptake.

Finally, since ours was a feasibility study with different models, we opted for a manganese dose range. However, since we cannot exclude that a 10% dose difference has no impact on results, in any future study aimed to dissect the performance of MEMRI, the use of an exact dose per weight would be recommended.

Immunohistochemistry analysis carried out on the pseudometastatic human prostate cancer animal model which progressed quickly (S1 Fig in S1 File), demonstrated high levels of expression of CaSR (score 4-5) either in the lung and intra-abdominal metastatic deposits. It has recently been investigated the role of CaSR in lethal prostate cancers, where higher CaSR tumour expression was associated with an approximately 2-fold higher risk for lethal progression, independently of Gleason grade and pathological stage [46]. However, in prostate or breast cancer, TRPV6 is definitively associated with highly proliferative and metastatic tumours [27, 28] thus, the combination of both highly expressed calcium receptors may have affected the different timing of tumour development and progression that we observed. Different distribution of the two calcium receptors within the tumours might also affect the type and level of manganese uptake observed at MRI. In this set of experiments, both receptors, CaSR and TRPV6, demonstrated similar distribution at immunohistochemistry analysis across the different type of tumour lesions. Certainly, a different distribution of both receptors is an indication of tumour heterogeneity as we observed in human studies [38] and this is important to take into account when we look at the translational potential of a study.

Finally, we also explored the possible relationship between the manganese signal enhancement observed at MRI and other cancer biomarkers typical of these tumour cell lines, such as hormone receptor status and ki67 (S1 Table in S1 File). MDA-MB-231 and PC3 cell lines demonstrated no correlation between CaSR or TRPV6 and hormone receptor or ki67 status and manganese uptake. Others applied MEMRI to investigate breast tumours and demonstrated that manganese uptake was correlated with cancer cell proliferation [43]. However, in this study calcium receptors expression level was not investigated.

The mechanism behind CaSR expression and activation is the result of a very complex combination of multi-receptor actions that affect the entire cellular calcium metabolism and thus, also cancer cell proliferation [47]. The level of extracellular calcium concentration is responsible of the activation of different intra and intercellular pathways which may affect the tumour microenvironment and, thus, the manganese uptake.

This is a preliminary study aimed to address the potential correlation between the different expression level of calcium receptors such as CaSR and TRPV6 and manganese uptake in different cancer animal models. Further studies need to explore more deeply the “active role” of calcium receptors on manganese uptake by using in vivo calcium blockers, as we have performed in our previous set of experiments [15]. Unfortunately, calcium blockers have important safety issues in preclinical experiments, particularly in pseudometastatic cancer animal models where their systemic administration could lead to death during the assay and the application is not allowed by the O.P.B.A. (Institutional Animal Welfare Body).

Further useful investigations aiming to demonstrate the “active role” of CaSR in MEMRI should take into consideration the knockdown/knock-in of CaSR in breast or prostate cancer cells. However, this type of experiment might very challenging due to unstable cancer features in the development of a metastatic breast or prostate cancer animal models, making the results obtained not reproducible.

To conclude the link between tumour and MEMRI is certainly interesting with high potential for further in vivo studies, necessary before considering its impact into the clinical setting. The link tumour/calcium receptors/manganese could provide functional information related to the high potential metastatic risk of breast or prostate tumours CaSR expressing. By using quantitative MEMRI, this work has demonstrated the potential of manganese for different types of breast or prostate tumour animal models in presence of different CaSR or TRPV6 level expression. For this reason, it needs further investigation considering the different and interesting routes that manganese can use to target tumours and one of these might be the presence of calcium receptors.

## Conclusions

Manganese enhanced Magnetic Resonance Imaging (MEMRI) was able to image human breast or prostate cancer animal models with different intra- and inter-tumour expression levels of CaSR. The expression level of TRPV6 did not relate to the Mn-uptake in MEMRI experiments.

## Materials and methods

### Human breast and prostate cancer cell lines

The human cancer cell lines PC3 (prostate cancer; ICLC) and MDA-MB-231 (breast cancer; Xenogen Corporation) were grown in RPMI 1640, with L-glutamine, 10% FCS and antibiotics (Lonza). Immunochemistry detection of calcium sensing receptor (CaSR) and Transient Receptor Potential channels 6 (TRPV6) was performed on sections of formaldehyde-fixed paraffin-embedded cell pellets, as described below for tissue samples.

### Human cancer animal models

All procedures involving animals have been approved by the Institutional Animal Welfare Body (O.P.B.A.) and complied with the national current ethical regulations regarding the protection of animals used for scientific purpose (D. Lvo, March 4, 2014, n. 26, legislative transposition of Directive 2010/63/EU of the European Parliament and of the Council of September 22, 2010 on the protection of animals used for scientific purposes).

Four six-week old female and two six-week old male NOD/SCID mice were obtained from a colony bred in house under sterile conditions. Different types of breast or prostate cancer animal models were respectively developed, as described below, as pilot experiments to test the suitability of the models.

### Human breast cancer orthotopic animal model

For the orthotopic breast cancer animal model, four six-week-old female, NOD-SCID mice were anesthetized with intraperitoneal injection of xylazine (10 mg/kg bw) - ketamine (100 mg/kg bw). MDA-MB-231 cells (1×10^6^ in 50 μl of serum free culture medium) were then injected in the lower left mammary fat-pad. The implantation was made under surgical sterile conditions. From 6 to 8 weeks later, when the tumour mass diameter was approximately 5 mm, as assessed by palpation and measurement with a calliper, mice were subjected to MR examination.

### Human prostate cancer orthotopic animal model

Two six-week-old male NOD-SCID mice were anesthetized as above and PC3 cells were implanted under surgical sterile conditions. The abdomen was cleaned with iodine solution and a 1 cm midline incision was made to expose the prostate gland. One million PC3 cells suspended in 50 μl of serum free culture medium were injected into a dorsal prostatic lobe using a 30-gauge needle. After 6 weeks, the mice were subjected to MR examination and when the tumour mass diameter was approximately 5 mm, MR with manganese injection was performed.

### Bone xenotransplant animal model of breast cancer

The osseous cancer model was set up according to Corey et al. [33]. Briefly, a small hole was drilled with a 30-gauge sterile needle through the tibia plateau, with the knee flexed, of the anesthetized mouse. Using a new sterile needle fitted to a 0.3 ml sterile syringe, a single-cell suspension of 1×10^5^ MDA-MB-231 cells in 10 μl was then carefully injected in the bone marrow cavity. After 6 weeks, the mice were subjected to MR examination and when the tumour mass diameter was approximately 5 mm, MR with manganese injection was performed. The progression of osteolytic lesions was monitored by X-Ray. During radiography examination under anaesthesia, the animals were laid in prone position and left sided position on a DIGITAL Mammography (IMS, Giotto Image). Post-processing analyses were done by a dedicated workstation (Barco Mio 5MP Led).

### Human prostate cancer animal model of metastatic disease

Prostate metastatic disease in animals was conducted as follows. Two five-week-old male, NOD-SCID mice were anesthetized with intraperitoneal injection of xylazine (10 mg/kg bw) - ketamine (100 mg/kg bw) and injected with 3×10^6^ PC3 prostate tumor cells by intracardiac route. After 6 weeks, the mice were subjected to MR examination to detect the presence of metastasis and then manganese injection was performed.

### Manganese enhanced Magnetic Resonance Imaging protocol

The pre-clinical MR imaging protocol was developed for an experimental mouse-dedicated volume coil with a diameter of 55 mm (linear birdcage transmit/receive coil, Flick Engineering Solutions BV-General Electric-Baio G.) applied to a clinical 3T MR system (Signa EXCITE®HDxT, GE, Milwaukee, USA). The MR protocol applied to these new cancer animal model experiments was as previously described [15].

As previously established [15], the manganese chloride solution (Sigma Chemical Co., St. Louis, MO, USA) was prepared (5 mM MnCl_2_ solution in 0.9% NaCl) and 180 μl per mouse were manually injected in the tail vein (corresponding to a dose range between 7.4 mg/kg in a 24 g mouse and 8.5 mg/kg in a 21 g mouse; animal weights were within the mentioned range). MR imaging was performed before and immediately after manganese administration was completed at the following time points 10, 30, 60 and 90 minutes, for the orthotopic cancer animal models (breast or prostate) and at 10, 30, 60, 90 minutes until 3 hours, for the metastatic cancer animal models (breast or prostate), in order to study the dynamics of manganese uptake per each metastatic lesion.

### MRI data and statistical analysis

MRI data were post-processed on a commercially available workstation (AW Volume Share 2, ADW 4.4, GE, Milano, Italy). The imaging sequences and parameters with the optimal signal noise to ratio (SNR) and signal enhancement (SE) relative to the manganese uptake were selected. Quantitative analyses were expressed as SE of each mouse. Tumour manganese quantification was performed by using a region of interest (ROI) which was drawn per each tumour (tumour dimeter was ranging from a minimum of 2 mm to a maximum of 10 mm). As reference organs for CaSR and TRPV6 expression, SE of muscle and kidney was compared with tumour SE, respectively [15, 26]. The SE values were divided by the background noise to yield the signal-to-noise ratio (SNR) according to the following formula:

SNR and SE% (calculated as [(SNR-SNR^0^)/ SNR^0^]×100) for all tumour lesions before and after manganese injection.

### Histopathological and immunohistochemistry analysis

Signs of animal distress and 1 cm maximum in tumour diameter were considered as endpoints of the study. Animal euthanasia by the carbon dioxide was performed shortly after the MRI session. At necropsy tumours were excised, fixed in buffered formalin (10%) and then paraffin-embedded for histological analysis as per standard techniques.

The sections with a thickness of 3 μm with the best representative areas of tumour tissue (>70%) and devoid of staining artefacts, (e.g. dye precipitates, tissue folds etc.) were identified by haematoxylin-Eosin staining and immunostained by using a BenchMark XT automated immunostainer (Ventana Medical Systems, Strasbourg, France). For bone histology, the limb was fixed in 10% buffered formalin and bones were fixed in 10% neutral buffered formalin, decalcified with a commercial chelating reagent for 2 hours and half, and trimmed for conventional paraffin embedding and histologic sectioning.

Calcium channel receptors status per each tumour sections was immunostained according to our previous developed protocol [15, 37, 38]. All the adequate areas in a section were analysed for staining pattern and intensity, to calculate the average expression level. The minimum number of adequate fields analysed per section was three. As the level of expression was consistent across the same tumour sample, one representative field was shown in figures.

The policlonal antibody antiTRPV6 was commercially available (abcam) with cytoplasmatic localization. Different diluitions were tested (1:500,1:1000,1: 3000) and the best one was 1:1000. The sections were deparaffinised, rehydrated and treated using the automatic immunostaininer Benchmark XT (Ventana Medical Systems, SA Strasbourg, France). Antigen retrieval was performed with citrate buffer high ph for 30 min. Then, the antibody was incubated for 1 h at 37°C followed by addition of the polymeric detection system (Ventana Medical System Ultraview Universal DAB Detection Kit). Finally, the sections were counter-stained (automatically) with Gill’s modified haematoxylin and then cover-s lipped in Eukit. As positive tissue control, placenta (Archived bank tissue, Department of Pathology, IRCCS Ospedale Policlinico San Martino, Genoa, Italy) was used. The sections were observed with a light microscope (Olympus, Tokyo, Japan) using progressive objectives (10X, 20X, 40X, 63X). Blind to the in vivo imaging results, two experienced pathologists (S.S. and M.T.) performed the qualitative analysis considering as parameters, the intensity and pattern of the staining for both calcium receptors (CaSR and TRPV6) classified on a 6-point scale (from score 0/absent expression to score 5/intense, widespread expression), as described in the literature [38].

## Supporting information

S1 File (DOCX)
