# Supplementary material for: Manganese-enhanced MRI (MEMRI) in breast and prostate cancers: Preliminary results exploring the potential role of calcium receptors
Source: PLoS One. 2020 Sep 15;15(9):e0224414. doi: 10.1371/journal.pone.0224414 (PMC7491733; doi:10.1371/journal.pone.0224414)
Supplement: S1 File — (DOCX) [file pone.0224414.s001.docx]

**
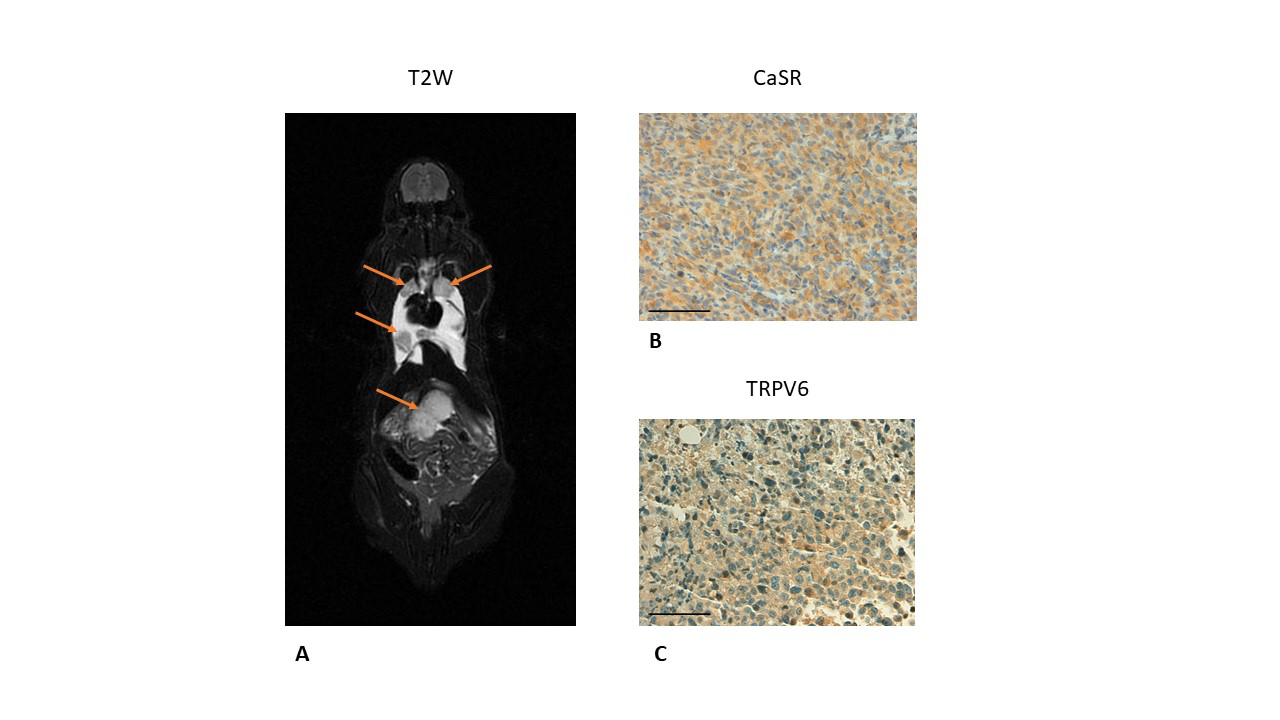
**

**S1 Fig. Ex-vivo MR imaging of pseudo-metastatic prostate cancer animal model and CaSR/TRPV6 receptors levels.** Tumor diameter was ranging from 4 mm to 10 mm. **A.** T2-weighted image of multiple metastatic deposits within the lungs and intra-abdominal (red arrows). Bilateral pleural effusion. **BC.** Immunohistochemistry (IHC) of CaSR and TRPV6 receptors displayed intense staining (score 4) in all the metastatic deposits in keeping with high aggressive prostate cancer. Scale bar=100µ.

**
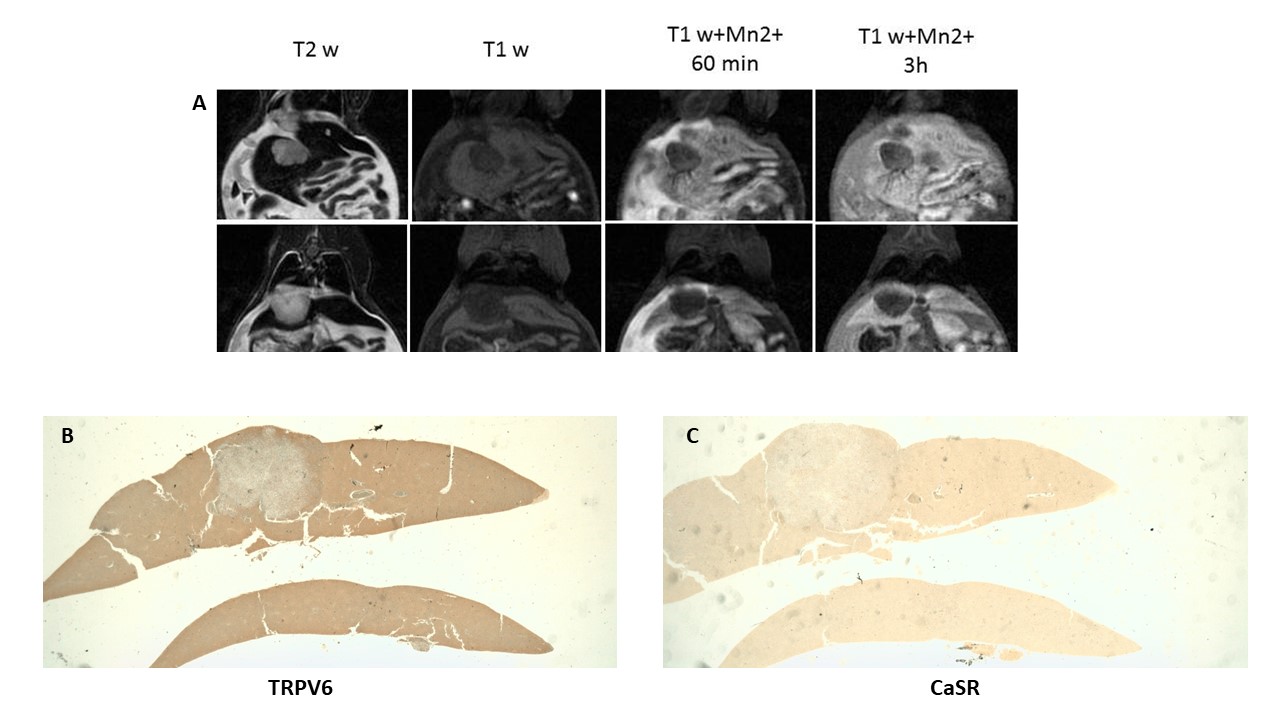
**

**S2 Fig. Manganese enhanced MR imaging (MEMRI) of pseudo-metastatic prostate cancer animal model and CaSR/TRPV6 receptors levels. A.** T2-weighted image of a liver metastasis. T1-weighted gradient echo images (T1 WI) recorded before and after 60 minutes and 3 hours of Mn2+ administration. No significant manganese uptake is appreciated, at both 60 minutes and 3 hours after Mn2+ administration. **BC.** Immunohistochemistry (IHC) of TRPV6 and CaSR: TRPV6 receptors displayed intense staining (score 4), while a rare positive staining of CaSR was detected in tumour cells (score 1). TRPV6 expression level in normal liver tissue was higher compared to CaSR expression level.

**In vivo MEMRI, calcium receptors expression and ki67 status.**

We also explored the possible relationship between the observed manganese-induced SE and other cancer biomarkers typical of these tumour cell lines, such as hormone receptor status and ki67. Immunohistochemically evaluation of Ki67, progesterone (Pgr) and estrogen (Er) receptors were performed by staining formalin fixed, paraffin-embedded 3-μm-thick tissue sections representative of the tumour previously identified through haematoxylin-eosin stained sections. (as per protocol described in the method section in the main text). MDA-MB-231 and PC3 cell lines, are respectively, triple negative (ER, PR and her2neu) and androgen receptor negative and our results showed, no significant correlation between CaSR or TRPV6 and hormone receptor or ki67 status (see results in Table 2 supplementary data) and manganese uptake. The possibility that the expression levels of CaSR or TRPV6 correlate with the cell density in the tumour tissue should be also considered. However, no major differences were apparent in the samples tested and the numerosity of our sample is not enough to reach significance for small differences.

**S1 Table.**

| **Xenotransplant lesions** | **CaSR**  **score** | **TRPV6**  **score** | **Ki67** | **SE**  **%** |
| --- | --- | --- | --- | --- |
| Orthotopic PC3 | 0 | 0 | 90 | +0.4 |
| Orthotopic PC3 | 1 | 1 | 5 | +7.5 |
| Orthotopic MDA-MB-231 | 0 | 0 | 80 | -2.0 |
| Orthotopic MDA-MB-231 | 4 | 4 | 43 | +91 |
| Intraosseous MDA-MB-231 | 0 | 0 | 98 | -7.5 |
|  |  |  |  |  |
| **Pseudo-metastatic lesions (PC3)** | **CaSR**  **score** | **TRPV6**  **score** | **Ki67** | **SE**  **%** |
| Liver nodule | 1 | 4 | 52 | -2.6 |
| Liver nodule | 1 | 4 | 52 | -5.6 |
| Mediastinal nodule | 3 | 2 | 70 | +33 |
| Mediastinal nodule | 2 | 2 | 70 | +17 |
| Diaphragmatic nodule | 3 | 4 | 70 | +47 |
| Peritoneal nodule | 1 | 1 | 47 | -0.4 |
| Peritoneal nodule | 1 | 1 | 47 | -0.9 |

**S1 Table.** SE% (calculated as [(SNR-SNR^0^)/ SNR^0^ ]×100) for all tumour lesions respectively, of xenotransplant and pseudometastatic tumour animal model, before and 90 minutes after manganese administration and CaSR, TRPV6 and Ki67 status of each lesion.
